# Association of delayed cord clamping with acute kidney injury and two-year kidney outcomes in extremely premature neonates: a secondary analysis of the Preterm Erythropoietin Neuroprotection Trial (PENUT)

**DOI:** 10.21203/rs.3.rs-4631779/v1

**Published:** 2024-07-19

**Authors:** Matthew Harer, Henry Zapata, Namrata Todurkar, Kristen Favel, Russell Griffin, Michelle Starr, Jennifer Charlton, Ryan McAdams, David Askenazi, Tapas Kulkarni, Shina Menon, Cherry Mammen

**Affiliations:** University of Wisconsin School of Medicine and Public Health; University of Florida School of Medicine -Jacksonville; University of California San Francisco; Indiana University School of Medicine; University of Wisconsin-Madison; University of Alabama at Birmingham; University of British Columbia

**Keywords:** delayed cord clamping, acute renal failure, chronic kidney disease, glomerular filtration rate, hypertension, long-term outcomes, prematurity

## Abstract

**Background:**

Delayed cord clamping (DCC) occurs in most preterm births.

**Objective:**

Evaluate the association of DCC with acute kidney injury (AKI) and two-year kidney outcomes.

**Methods:**

Secondary analysis of the Preterm Erythropoietin Neuroprotection Trial of neonates born 24^0/7^ to 27^6/7^ weeks’ gestation. AKI and two year kidney outcomes were compared in neonates with DCC (≥30 seconds after delivery) to those with early cord clamping (ECC) (<30 seconds after delivery).

**Results:**

The incidence and severity of AKI did not differ between the DCC and ECC groups (aOR 1.17 [95%CI 0.76–1.80]). At two years corrected age, DCC was associated with a 4.5-fold times increased adjusted odds of eGFR <90 mL/min/1.73m^2^. No significant associations were noted between DCC and albuminuria or elevated BP.

**Conclusions:**

DCC was not associated with decreased neonatal AKI, but was associated with higher adjusted odds of eGFR <90 mL/min/1.73m^2^ at two years.

## Introduction

In the 20th century, early cord clamping (ECC) became standard obstetrical practice to reduce maternal post-partum hemorrhage risk. However, over time evidence has accumulated regarding the benefits of delayed cord clamping (DCC) for the newborn. By the early 2000’s, international medical organizations began recommending DCC in vigorous newborns as the standard of care. [1–2] Newborns undergo multiple complex physiological transitions in the first few minutes after birth, including changes to neonatal respiratory and circulatory systems. [3] By allowing additional placental blood transfer to the neonate, DCC facilitates the postnatal transition by increasing blood volume, oxygenation, number of red blood cells, cardiac output, and iron delivery. [4–5] DCC is now associated with reduced mortality and has therefore emerged as a standard of care for all gestational ages. [5, 6] In premature neonates, DCC is associated with a decreased risk for intraventricular hemorrhage (IVH), necrotizing enterocolitis (NEC), and sepsis compared to ECC. [7–8] There is also evidence suggesting DCC improves short and long-term neurodevelopment in preterm infants. [9–11]

Limited data exist on the association of DCC and kidney outcomes, especially in extremely low gestational age neonates (ELGANS). Cord management practices may contribute to changes in short and long-term kidney outcomes through a variety of mechanisms. The stabilization of the renin-angiotensin-aldosterone system [12–14], improved systemic vascular resistance through decreased fluctuations in systemic blood pressure [15–17], transfer of growth factors, progenitor cells, or stem cells [10, 18] may enhance nephrogenesis and result in improved kidney growth and function in those receiving DCC. It may offer further advantages by enhancing neonatal hemodynamic stability and hematologic indices thus improving neonatal kidney perfusion and oxygenation. [19] A retrospective single-center cohort study of 278 very low birth weight neonates found no differences in acute kidney injury (AKI) by cord management strategy and no studies have compared two year kidney outcomes by cord management strategy. [20]

Our objective was to evaluate the association of different cord management strategies with AKI and two-year kidney outcomes in ELGANs. We hypothesized that in ELGANs, DCC would be associated with decreased in-hospital AKI and improved two-year kidney outcomes compared to ECC.

## Methods

### Study population

This study was a *post hoc* secondary analysis of the Preterm Erythropoietin Neuroprotection trial (PENUT) (ClinicalTrials.gov Identifier: NCT01378273), a multicenter, randomized, double-blind trial conducted between December 2013 and September 2016 in ELGANs born 24^0/7^ to 27^6/7^ weeks of gestation who received either high-dose erythropoietin or placebo. [21] Kidney outcomes, both short and long-term have been previously reported in the ancillary Recombinant Erythropoietin for Protection of Infant Renal Disease (REPAIReD) study, to investigate kidney outcomes in ELGANs. [22] The PENUT study was approved by local institutional review board of each participating center. Informed written consent was obtained from the parent or legal guardian.

Data on maternal and neonatal baseline characteristics including race, ethnicity and neonatal medication exposure were collected as part of the PENUT study. [21] Urine, blood, and blood pressure (BP) measurements were not mandatory during follow-up, but were encouraged as part of the REPAIReD study. [22–23] Further details on design, definitions of demographic factors, comorbidities, primary efficacy, and safety outcomes have been previously published. [21, 24]

For this secondary analysis, we included all ELGANs randomized in PENUT with data on cord management available. For the analysis of AKI outcomes, we excluded neonates who died in the first two days of life and those with insufficient serum creatinine (sCr) data to determine the presence of AKI. For two-year outcomes, we included subjects with estimated glomerular filtration rate (eGFR), urine albumin/creatinine ratio (ACR), or BP measured at the two-year visit. We excluded those with death prior to 24 months, no follow-up, no blood, urine, or BP measured.

### Definitions of variables:

Cord clamping data was obtained as part of the PENUT study. DCC was defined as cord clamping performed 30 seconds or more after delivery, and ECC was defined as cord clamping performed less than 30 seconds after birth. [21] AKI was defined by Kidney Disease Improving Global Outcomes (KDIGO) criteria based on sCr measurements, consistent with other studies from this cohort. [21, 25–26] Baseline sCr was determined as the lowest prior sCr measurement after the first 48 hours of life, excluding the initial two days to avoid maternal sCr influence. [23, 27–29] Severe AKI was defined as stage 2 or 3. Urine output was not included in the AKI definition as it was not recorded uniformly across sites. Similar to previously published studies in this cohort, AKI was stratified into early (days 3–7) and late (> 7 days) onset. [27]

We evaluated three a *priori* designated kidney outcomes at two years [22, 30]: 1) eGFR < 90 ml/min/1.73m^2^ using the CKID U25 equation (including sCr and cystatin C) [31–32], 2) albuminuria, defined as ACR > 30 mg albumin/g creatinine [33], and 3) systolic or diastolic BP > 90th percentile for age and sex based on 2017 AAP guidelines. [34–35] Urine samples were collected using a bag specimen or a cotton ball in the diaper. BP readings were obtained using a Briggs Mabic Healthcare Manual Sphygmomanometer with an appropriately sized BP cuff, measured twice with a five-minute interval between readings. The lowest systolic BP (sBP) and diastolic BP (dBP) were recorded [22–23]. As a *post-hoc* analysis, eGFR was analyzed as a continuous outcome.

### Statistical Analysis

Maternal demographics and neonatal characteristics between groups were summarized using descriptive statistics. Median and interquartile range were used to report when the data were not normally distributed or for discrete data (Apgar scores). Mean and standard deviation are reported for data with normal distribution. We used a generalized estimating equation (GEE) logistic regression model (to account for potential clustering by study site) to evaluate AKI outcomes between DCC and the association between DCC and two-year kidney outcomes. Adjusted models were created by using PROC HPGENSELECT in SAS (SAS Institute, Cary, North Carolina, USA) applying a grouped least angle selection and shrinkage operator (LASSO) selection [36] to the logistic models for each separate outcome. For the two-year outcomes (i.e., eGFR < 90 ml/min/1.73m^2^, ACR > 30 mg albumin/g creatinine, and BP > 90th percentile), adjusted odds ratios are reported for the outcome’s individual LASSO-selected model in addition to an adjusted model that includes all covariates from the LASSO selection of the three outcomes (for purposes of qualitatively comparing associations across the outcomes). For AKI outcomes, adjusted models included gestational age, sex, small for gestational age, surfactant use during delivery room resuscitation, blood transfusion, BPD at 36 weeks, NEC, patent ductus arteriosus (PDA), severe sepsis, severe IVH, non steroidal anti-inflammatory drug (NSAID) exposure before AKI, vancomycin exposure before AKI, and vasopressor medication exposure before AKI. For two-year outcomes, adjusted models included gestational age, maternal pre-eclampsia, small size for gestational age, vancomycin exposure, vasopressor exposure, maternal race, surfactant use during resuscitation, sex, and PDA. Statistical significance was defined as p < 0.05.

## Results

### Patient Characteristics

**Figure 1:**
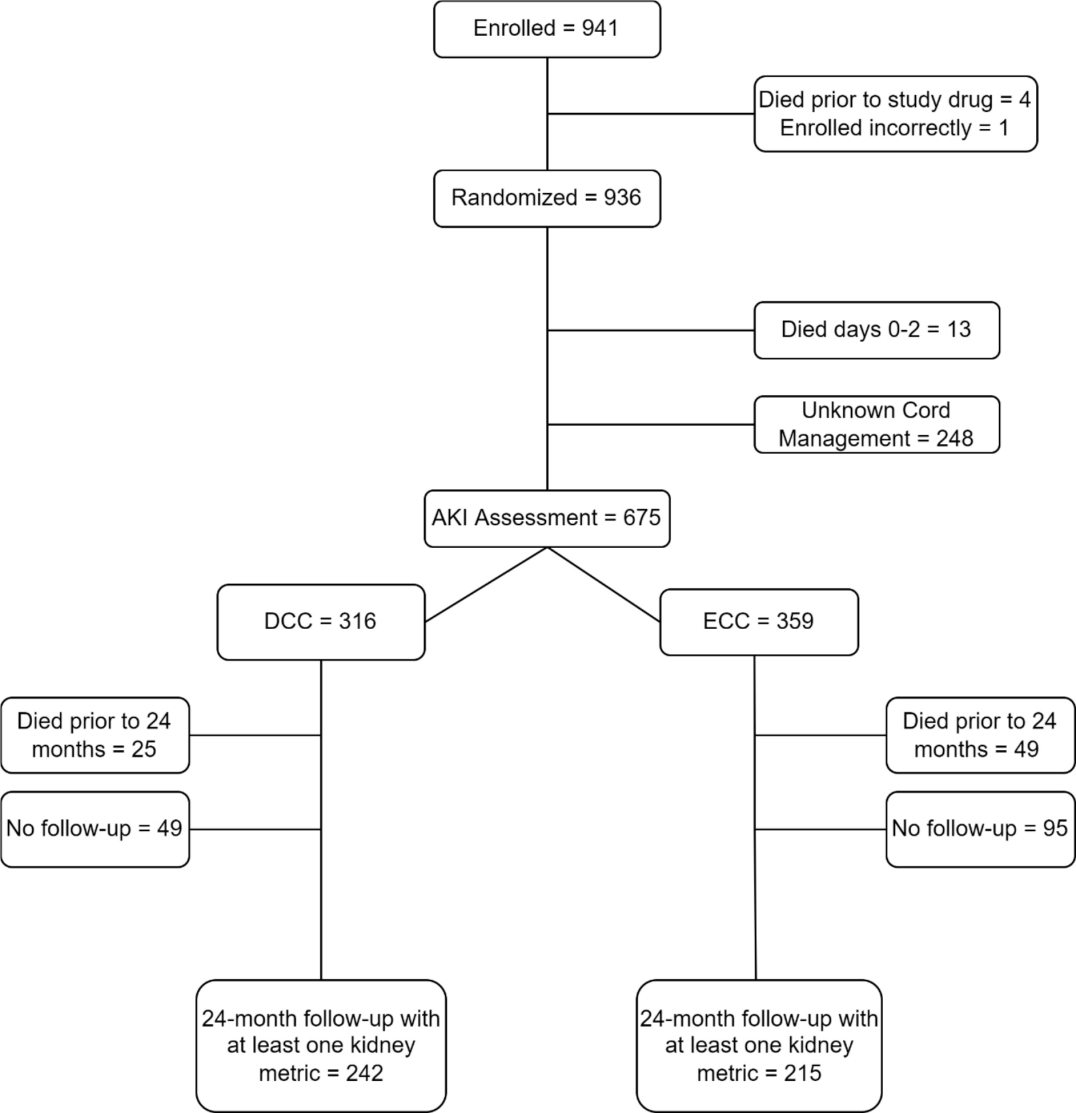
CONSORT diagram

Of the 936 neonates randomized, 13 died in the first two days of life, and 248 neonates did not have cord management recorded. Therefore, our secondary analysis for AKI included 675 ELGANs with documented cord management data (DCC n = 316, ECC n = 359) (Fig. 1).

Cohort characteristics by cord management approach are summarized in Table 1. Birthweight was higher in the DCC compared with the ECC group (821 vs. 789 g, p = 0.02). DCC was performed less frequently in Black neonates compared to White neonates (33 vs. 53%, p < 0.01). Hispanic neonates were more likely to have DCC than non-Hispanic neonates (64 vs. 42%, p < 0.01). The DCC group had higher median Apgar scores at 1 and 5 minutes (4 vs. 3, p = 0.02 and 7 vs. 6, p < 0.01 respectively), greater exposure to 2 doses of prenatal steroids (72% vs. 60%, p < 0.01), less exposure to vasopressor medications (37.3% vs 46.8%, p < 0.01), less exposure to indomethacin/ibuprofen (45% vs 67%, p < 0.01) but similar vancomycin (60% vs 64%, p = 0.26) and gentamicin (95% vs. 97%, p = 0.26) exposure prior to AKI. About 36% of infants received gentamicin for 48 hours, 25% for 72 hours and 11% for a full course of 7 days. The DCC group had higher hematocrit values at birth and at 48 hours, and they were less likely to need a blood transfusion (73% vs 85%, p < 0.01).

### Acute Kidney Injury

**Figure 2:**
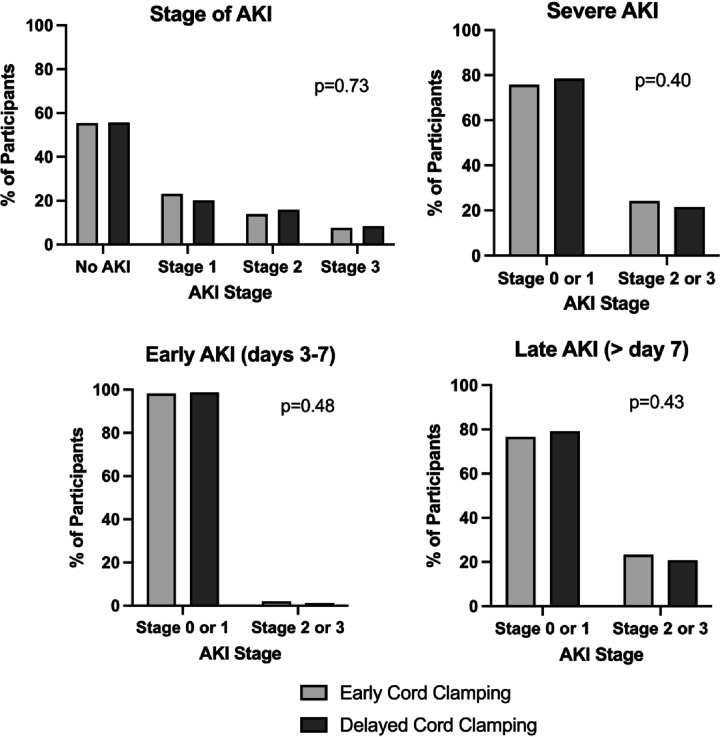
AKI outcomes No differences in crude rates of any, severe, early, or late acute kidney injury between delayed cord clamping and early cord clamping groups.

Overall, 45% of neonates had AKI and 23% had severe AKI (stage 2 or 3) in the hospital. There were no differences in crude rates of any, severe, early, or late AKI between DCC and ECC groups (Table 2, Fig. 2). After multivariable adjustment, no differences in AKI rates by cord management strategy were identified for any stage AKI (aOR 1.17 [95%CI 0.76–1.80]) or severe AKI (aOR 0.97 (95%CI 0.61–1.53)) (Table 3).

### Two-Year Kidney Outcomes

**Figure 3:**
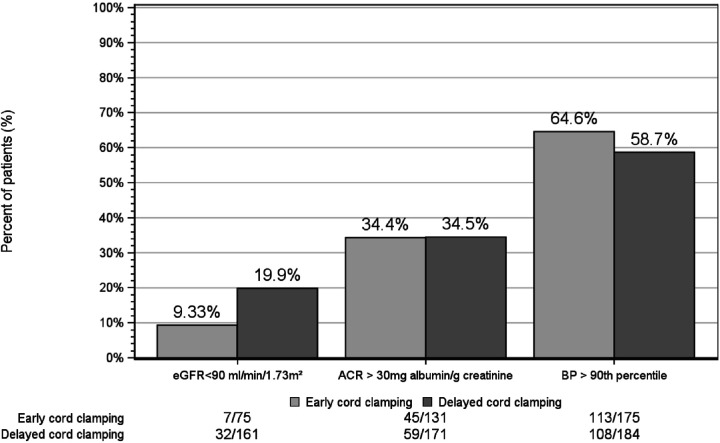
Comparison of two-year kidney outcomes by cord clamping status

After excluding those who died and those who did not have a follow-up kidney outcome measured, the sample size for our two-year kidney outcome cohort was 457 infants (DCC n = 242, ECC n = 215, Fig. 1). At 22–26 months’ corrected gestational age, 20% (n = 32) of DCC and 9% (n = 7) of ECC infants had an eGFR < 90 mL/min/1.73m^2^. There were no statistically significant differences in height or weight between the two groups of patients (84.5 vs. 84.7 cm (p = 0.61), 11.5 vs. 11.7kg (p = 0.2)). Albuminuria was seen in 34% (n = 59) of DCC and 34% (n = 45) of ECC infants, and systolic and/or diastolic BP > 90th percentile was observed for 59% (n = 108) of DCC and 65% (n = 113) of ECC infants (Table 4, Fig. 3).

**Figure 4:**
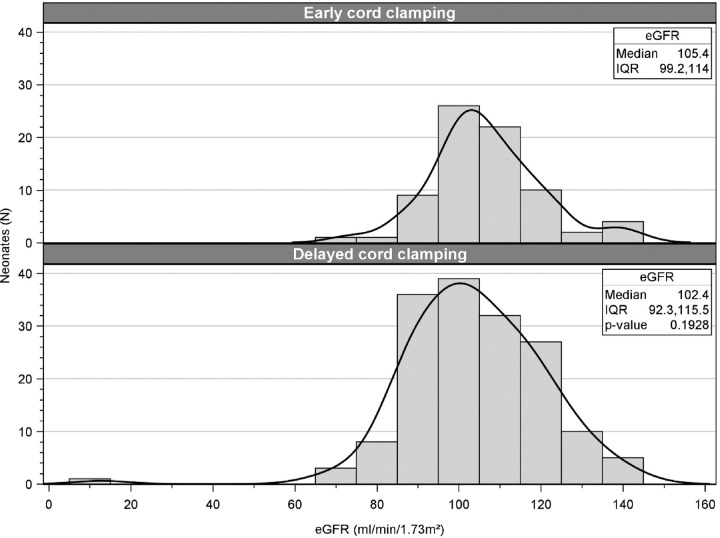
Distribution of GFR in ECC v/s DCC groups Histogram by both cord clamping techniques provide comparison of distributions of continuous eGFR. The bars are in 10-unit increases (e.g., 80 includes those with an eGFR of 80–89, 100 includes those from 100–109).

In adjusted models, no association was observed between DCC and ECC infants in regard to albuminuria (aOR 1.15 [95%CI 0.82–1.62]) or BP > 90th percentile (aOR 0.84 [95%CI 0.57–1.25]); however, an over four-fold increased odds of eGFR < 90 ml/min/1.73m^2^ observed among DCC infants (aOR 4.56 [95%CI 1.18–17.63]). A histogram by cord clamping management with kernel density line provides a comparison of the distribution of continuous eGFR, shown in Fig. 4. In the post hoc analysis of continuous eGFR outcomes, no statistical difference was seen between ECC vs. DCC groups (median 105.4 (IQR) vs. 102.4(IQR) respectively, Wilcoxon rank sum p = 0.1928). Although greater number of infants in DCC had eGFR data available (51% vs. 21%), the distribution of eGFR values were comparable in the DCC and ECC neonates.

## Discussion

In this secondary analysis, we compared the kidney outcomes in ELGANs by umbilical cord management strategies after birth. We did not see a decreased rate of AKI nor improved 2-year kidney outcomes in those with DCC as we hypothesized. DCC was associated with an increased rate of eGFR < 90mL/min/1.73m^2^ at two years compared to ECC. After adjusting for confounding factors, infants with DCC were over four times as likely to have decreased eGFR at two years compared to ECC. However, when evaluating eGFR as a continuous variable as a post hoc analysis instead of a dichotomous outcome, this association was no longer significant.

In our study, despite demographic differences in the groups that indicate the DCC group may have lower risk of AKI, such as birth weight and Apgar scores, the DCC group did not have lower rates of AKI by timing or stage of severity. Our findings are consistent with previously published data. [20] There are a few potential explanations for our findings. First, in a separate evaluation of this same cohort, it was found that correcting sCr for fluid balance uncovered numerous missed episodes of AKI in the cohort. [43] It is possible that these missed episodes of AKI when correcting for fluid balance could alter the distribution of AKI between the groups and lead to different findings through misclassification. Second, it is possible that since AKI in this study was only defined by sCr that cases of AKI defined by urine output were missed and could have resulted in differences in AKI rates by group. Oh and colleagues evaluated a group of term female neonates and found that, in the first 12 hours after birth, DCC neonates had higher urine output compared with ECC group, which makes it possible that not accounting for urine output defined AKI may result in further misclassification. [44] A prospective study that defines duration of delayed cord clamping, monitoring of urine output in addition to sCr, as well as urinary biomarkers of kidney injury may help to understand differences in AKI by cord management strategies.

With regards to two-year kidney outcomes, our findings raise questions about the association between cord management style and kidney function during childhood in former ELGANs. Although we defined our two-year eGFR outcome a priori as a dichotomous outcome, we performed additional *post-hoc* evaluation as a continuous outcome to further understand our results. While eGFR as a dichotomous variable resulted in significant differences between groups, the continuous eGFR evaluation did not detect any differences. Notably, the only subject who had eGFR < 20 ml/min/1.73m^2^ was in the DCC group – which could skew the data while analyzing eGFR as continuous variable. We believe that the dichotomous outcome is important to report as eGFR < 90 ml/min/1.73m^2^ is the cut-off for chronic kidney disease (CKD) in this population. However, we recognize that the continuous results align more with the lack of differences seen in our proteinuria and hypertension outcomes. These conflicting results create equipoise as to how DCC affects two-year kidney function in former ELGANS.

There are several plausible explanations for our findings that an eGFR < 90 mL/min/1.73m^2^ (as a dichotomous outcome) at two years is independently associated with DCC. First, it’s possible that indeed DCC impacts kidney development and two-year CKD rates. Second, although we performed analyses to control for potential confounding, it is possible that other measured or unmeasured factors or interactions were not adequately controlled. We were unable to account for post-discharge factors such as nutrition, medications, and possible additional AKI episodes that could affect two year kidney outcomes [37–38] Furthermore, the effect of prenatal steroids on nephrogenesis, nephron endowment, and kidney function may in part account for our findings on eGFR, and although we adjusted for antenatal steroid exposure, those in the DCC group had higher rates of antenatal steroid exposure. Antenatal steroid administration has been shown in preclinical models to have a direct effect on fetal renal blood flow, functional glomerular surface area available for filtration, and glomerular filtration. [39–41] Observational studies and preclinical data suggest antenatal steroid exposure is associated with poor cardiovascular outcomes via numerous mechanisms, including programming of the renin-angiotensin-aldosterone system. [42] Studies on pregnant sheep and their offspring have shown that antenatal steroids are associated with fewer glomeruli independent of fetal kidney weight, suggesting that antenatal steroid exposure affects nephron development and possibly kidney function later in life. [42] Further analyses of other populations and past studies would be needed to understand this complex relationship as there is not equipoise at this time to perform a randomized trial of either antenatal steroids or DCC in this population given the overall positive outcomes and improvements in other organ systems.

We note that in our cohort, a higher proportion of Black and non-Hispanic ELGANs were managed with ECC. Racial disparities in the NICU have persisted for decades, especially in vulnerable populations such as preterm infants. Black infants are at risk of lower health care access, residential segregation, and poverty. [45–46] Historically, non-Hispanics have been reported to receive better care, but in our study babies with maternal Hispanic ethnicity were more likely to receive DCC. [47] Further large multicenter studies are needed to delineate the proportion of birthing mothers who are receiving DCC and what factors are leading to these differences.

This study has several strengths, including the use of high-quality, prospectively collected data from a large multicenter randomized trial of ELGANs which provides a robust data source to analyze research questions related to kidney outcomes. However, there are several limitations. Given our study design, we can only present observational associations rather than causation. Secondly, a substantial proportion of participants did not have kidney assessment at the two-year follow-up, which reduces the power to detect outcomes and raises the potential for bias in the results. However, analyses of this cohort have shown that no significant differences in baseline characteristics were found between those with and without follow-up assessment. [29] In addition, a sensitivity analysis of this cohort reported no evidence of potential bias resulting from unequal representation due to patient loss to follow-up status.[23] Furthermore, in this study urine output was not measured so there may have been a higher incidence of AKI than was reported and different distribution between that groups that could change our results.[23, 48] Given the retrospective, secondary analysis nature of this study, it was likely underpowered to detect smaller, but potentially clinically meaningful differences between cord clamping groups. Although we used the most contemporary definition of eGFR, this measure is not precise and was not developed specifically for children less than two years.

In conclusion, in this secondary analysis of ELGANS, we did not see any differences in neonatal AKI rates by cord clamping approach. However, DCC was associated with increased rates of eGFR < 90 mL/min/1.73m^2^ at two years compared to ECC, a finding which persisted after multivariable adjustment, but was not present on continuous evaluation of eGFR. Further research to quantify the impact of cord clamping strategies and prenatal steroids on short and long-term kidney outcomes in this vulnerable population is needed. Potential future studies could evaluate novel markers of kidney injury (such as renal near-infrared spectroscopy, renal microcirculation, bioenergetics [oxidative stress, mitochondrial function], urine output, and other biomarkers [such as urinary neutrophil gelatinase-associated lipocalin]) in patients receiving different cord clamping techniques. Prospective long-term follow-up studies are needed of kidney function in preterm neonates that include data on cord management as well as childhood exposures to validate the association between DCC and reduced eGFR at two years of age.

## Figures and Tables

**Table 1:** Demographics

	Delayed Cord Clamping n = 316 (n%)	Early Cord Clamping n = 359 (n%)	p-value*
**Gestational Age**			0.08
24 Weeks	74 (23.4)	84 (23.4)	
25 Weeks	90 (28.4)	92 (25.6)	
26 Weeks	63 (19.9)	100 (27.9)	
27 Weeks	89 (28.1)	83 (23.1)	
**Sex**			0.57
Female	153 (48.4)	166 (46.3)	
Male	163 (51.6)	193 (53.8)	
**Maternal Race**			< 0.01
Black/African American	56 (17.7)	113 (31.5)	
Other/unknown	25 (7.9)	39 (10.9)	
White	235 (74.4)	207 (57.7)	
**Maternal Ethnicity**			< 0.01
Hispanic/Latino	95 (30.0)	54 (15.0)	
Not Hispanic/Latino	220 (69.6)	299 (83.3)	
Unknown	1 (0.3)	6 (1.7)	
**Size**			
Normal or Large for Gestational Age	273 (86.6)	297(82.9)	0.18
Small for Gestational Age	42 (13.3)	61 (17.0)	
Birth weight, g, mean (SD)	821 (183)	789 (185)	0.02
Birth length, cm, mean (SD)	33.0 (3.0)	32.7 (2.8)	0.25
Occipitofrontal circumference, cm, mean (SD)	47.6 (1.7)	47.3 (2.7)	0.28
Apgar 1 min, median (IQR)	4 (2,6)	3 (2,6)	0.017
Apgar 5 min, median (IQR)	7 (6.8)	6 (5,8)	0.002
**Prenatal steroids, n (%)**			< 0.01
None	15 (4.7)	45 (12.5)	
1 dose	49 (15.5)	68 (18.9)	
2 doses	228 (72.2)	216 (60.2)	
3 doses	24 (7.6)	30 (8.4)	
**Delivery Room Resuscitation**			
Any	311 (98.4)	350 (97.4)	0.4
Intubation	240 (75.9)	302 (84.3)	0.007
Surfactant	158 (50.0)	193 (53.9)	0.33
Chest compressions	21 (6.6)	32 (8.9)	0.27
**Cord Clamping outcomes**			
Hematocrit (birth), mean (SD)			
Low	43.6 (6.2)	42.0 (6.1)	0.002
High	44.4 (6.2)	42.9 (6.1)	0.003
Hematocrit (48h), mean (SD)			
†Low	39.4 (6.8)	37.3 (6.5)	0.0005
†High	40.7 (6.2)	38.2 (5.8)	< 0.01
# of Blood Transfusions < 28d, mean (SD)	3.0 (3.1)	3.6 (3.1)	0.0069
Blood transfusion, n (%)	232 (73.4)	305 (85.0)	0.0002
**Infant Exposures**			
Vasopressor Medications	118 (37.3)	168 (46.8)	0.01
Nephrotoxic Meds			
Indomethacin/Ibuprofen	142 (44.9)	239 (66.6)	< 0.01
Gentamicin	300 (94.9)	347 (96.7)	0.26
No gentamicin	12 (3.3)	16 (5.0)	
<72h	91 (25)	59 (18.6)	
>72h	256 (71.3)	241 (76.2)	
Vancomycin	190 (60.1)	231 (64.3)	0.25

* Estimated from a chi-square test for categorical and a t-test or Wilcoxon rank sums test for continuous variables

† The lab values are represented as high for the day and low for the day.

**Table 2:** AKI outcomes

	Delayed Cord Clamping n = 316 (%)	Early Cord Clamping N = 359 (%)	*P* value*
AKI Max anytime, n (%)			0.73
Stage 0	175 (55.4)	200 (55.7)	
Stage 1	73 (23.1)	72 (20.1)	
Stage 2	44 (13.9)	57 (15.9)	
Stage 3	24 (7.6)	30 (8.4)	
Severe AKI max anytime, n (%)			0.40
No (stage 0 or 1)	248 (78.5)	272 (75.8)	
Yes (stage 2 or 3)	68 (21.5)	87 (24.2)	
Early (days 3–7), n (%)			0.48
Stage 0/1	312 (98.7)	352 (98.1)	
Stage 2/3	4 (1.2)	7 (1.9)	
Late (> day 7), n (%)			0.43
Stage 0/1	250 (79.1)	275 (76.6)	
Stage 2/3	66 (20.8)	84 (23.3)	

* Estimated from a chi-square test

**Table 3:** Logistic Regression – AKI outcomes

	Early Cord Clamping (n = 359)	Delayed Cord Clamping (n = 316)		
AKI Outcome	N (%)	N (%)	Crude* OR (95% CI)	Adjusted† OR (95% CI)
Any AKI	159 (44.3)	141 (44.6)	1.01 (0.68–1.51)	1.17 (0.76–1.80)
Severe AKI	87 (24.2)	68 (21.5)	0.86 (0.58–1.27)	0.97 (0.61–1.53)

* Estimated from a general estimating equation logistic regression clustered by study site

† Adjusted for gestational age, sex, small for gestational age, surfactant use during delivery room resuscitation, blood transfusion, BPD at 36 weeks, NEC, PDA, severe sepsis severe IVH, NSAID exposure before AKI, vancomycin exposure before AKI, and vasopressor medication exposure before AKI

**Table 4:** 22–26 Month Kidney Outcomes

24-month Outcome	DCC N (%)	ECC (%) N (%)	Crude* OR (95% CI)	Adjusted† OR (95% CI)	Adjusted‡OR (95% CI)
**OVERALL**					
eGFR < 90 ml/min/1.73m^2^	32/161 (19.9)	7/75 (9.3)	2.41 (1.01–5.75)	4.22 (1.18–15.09)	4.56 (1.18–17.63)
ACR > 30 mg albumin/g creatinine	59/171 (34.5)	45/131 (34.4)	1.01 (0.62–1.63)	1.14 (0.80–1.63)	1.15 (0.82–1.62)
BP > 90th percentile	108/184 (58.7)	113/175 (64.6)	0.78 (0.51–1.19)	0.77 (0.52–1.16)	0.84 (0.57–1.25)

* Estimated from a general estimating equation logistic regression clustered by study site

† Adjusted for: eGFR outcome: gestational age, maternal pre-eclampsia, small for gestational age, vancomycin exposure, and vasopressor exposure ACR outcome: maternal race and surfactant use during resuscitation BP outcome: sex, surfactant use in resuscitation, PDA

‡ Adjusted for gestational age, maternal pre-eclampsia, small for gestational age, vancomycin exposure, vasopressor exposure, maternal race and surfactant use during resuscitation, sex, and PDA

Abbreviations: DCC, delayed cord clamping; ECC, early cord clamping; OR, odds ratio; eGFR, estimated glomerular filtration rate; ACR, albumin creatinine ratio; BP, blood pressure
